# Supplementary material for: Dexterous electrical-driven soft robots with reconfigurable chiral-lattice foot design
Source: Nat Commun. 2023 Aug 21;14:5067. doi: 10.1038/s41467-023-40626-x (PMC10442442; doi:10.1038/s41467-023-40626-x)
Supplement: Supplementary file 1 — Supplementary Information [file 41467_2023_40626_MOESM1_ESM.pdf]

Supplementary Materials for
"Dexterous electrical-driven soft robots with reconfigurable chiral-lattice foot design"

Dong Wang[1, 2*†], Baowen Zhao[1, *], Xinlei Li[1], Le Dong[1], Mengjie Zhang[1], Jiang Zou[1], Guoying Gu[1,2 †]

[1]State Key Laboratory of Mechanical System and Vibration, School of Mechanical Engineering, Shanghai Jiao Tong University, Shanghai 200240, China
[2]Meta Robotics Institute, Shanghai Jiao Tong University, Shanghai 200240, China

*These authors contributed equally to this work.

†Corresponding author. Email: wang_dong@sjtu.edu.cn (D.W.); guguoying@sjtu.edu.cn (G.Y.G)

Supplementary Note 1. Structural design

1D, 2D and 3D chiral lattices are designed (Supplementary Fig. 1a-c). The lattices are formed by connecting 1D, 2D or 3D microstructures with the top and bottom plates. The microstructures are straight, horseshoe, or spiral structures, respectively. To form a chiral twisting effect, the top plate is rotated by an angle γ counter-clockwise. The magnitudes of the parameters in Supplementary Fig. 1 are L = 12.45 mm and w = 1.5 mm in Supplementary Fig. 1a, L =13.95 mm, R = 1.975 mm in Supplementary Fig. 1b and L = 15 mm, w = 1.5 mm, R = 2 mm, d = 5 mm in Supplementary Fig. 1c. The FE simulated axial strain and the twisting angle of the top plate α as a function of γ under a fixed compression force 0.003N are shown in d and e. α increases with γ, indicating a chiral twisting effect. The 3D lattice foot shows a significantly larger axial strain (larger than 100%), compared to 1D and 2D lattice feet. The dependences of the axial displacement and the twisting angle on the applied force F are shown in f and g. γ ranges from 0 to 90º.

The dimensions of the soft robots are given below. The plain foot is a cuboid with length, width and thickness of 20 mm, 20 mm and 1 mm, respectively. The design parameters of the chiral lattice foot are shown in Supplementary Fig. 2. Both left- and right-handed chiral lattice foot are used in this work. When demonstrating the turning motions, only left-handed chiral lattice foot is used. For the lattice foot, the upper surface of the foot is a square with a side length D = 15 mm. The height H = 2 mm. The lattice foot consists of 8 straight beams. The length L = 12.45 mm and diameter w = 1.5 mm. All the beams are twisted with γ = 90 °. A triangular prism is located at the bottom of the lattice foot with α = 7°, b = 2.45 mm. The CAD design of the acrylic frame is shown in Supplementary Fig. 3. The thickness of the acrylic sheet is 0.3mm.

The dependence of the largest twisting angle α under different frequencies f is plotted in Supplementary Fig. 4a by combining the experimental and theoretical methods. We first calculate the maximum Δz at the steady state under various f (Supplementary Fig. 4b). Supplementary Fig. 4c compares the FEM simulated and experimental α and Δz. A reasonable agreement is achieved. The relationship between α and f is then obtained using Supplementary Fig. 4b and c. We can observe that the largest twisting angle decreases slightly from ~7.6º to ~4.7º when f increases from 30 Hz to 70 Hz.

We measured the rotation curvature of the soft robot as a function of the frequency, as shown in Supplementary Fig. 4d. We can observe that the radius decreases significantly when the applied frequency decreases from 40 to 50 Hz. When the applied frequency reaches 50 Hz, the rotation of the soft robot is almost in position. This phenomenon can be explained below. The radius depends on both the twisting angle and forward speed. The forward speed decreases significantly when the frequency increases due to the bandwidth and creep of the DE actuator. Supplementary Movie 11 shows the turning motion of the soft robot under f = 54 Hz. We can observe that the soft robot twists almost at its original position. The saddle shape DE actuator keeps at an expanded state at high frequency, which cannot provide enough actuation force for the forward motion.

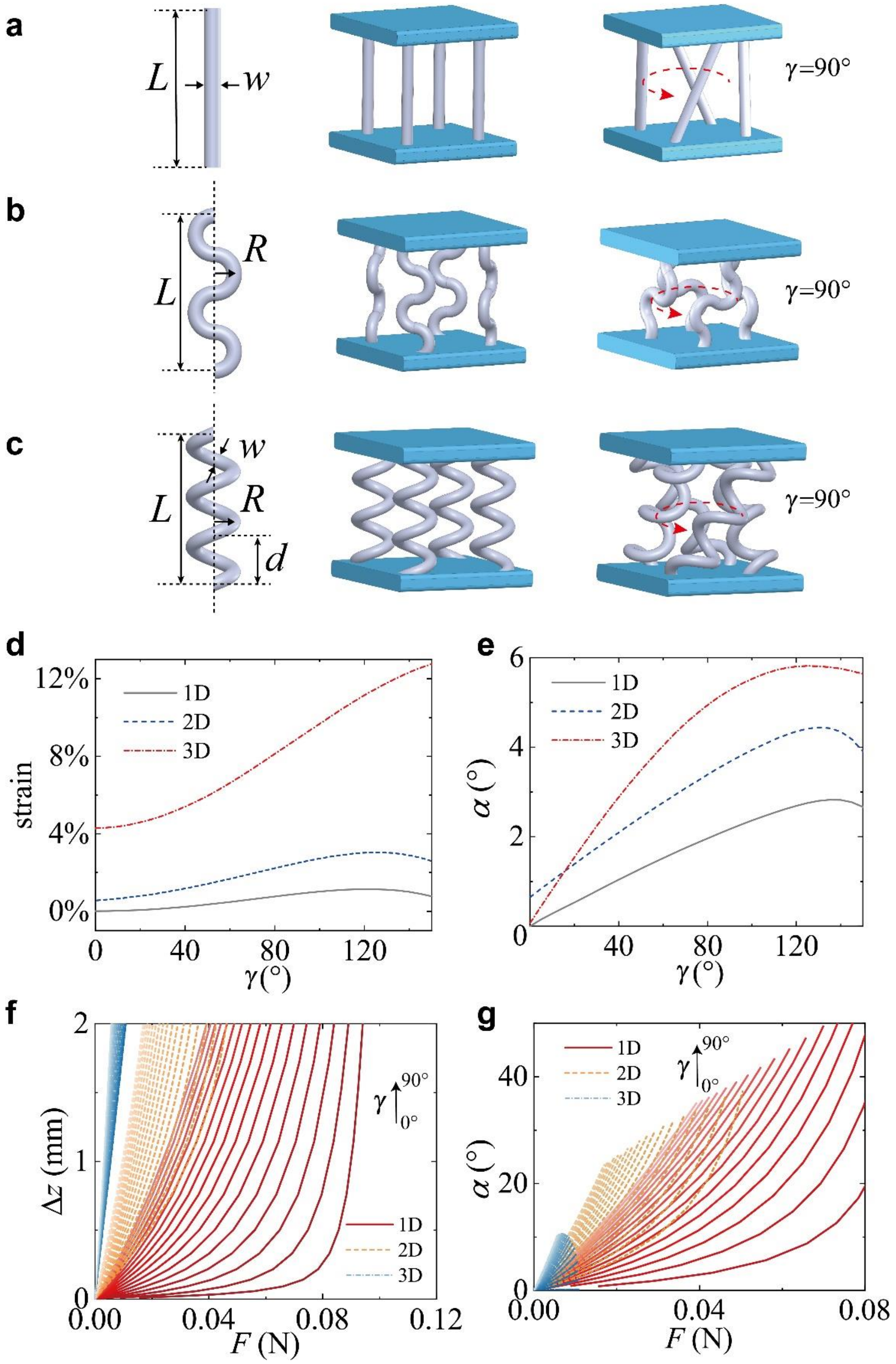

Supplementary Fig. 1. Design of 1D, 2D and 3D chiral lattices. 1D, 2D and 3D chiral lattices are designed **a-c**. The FE simulated axial strain and the twisting angle of the top plate α as a function of γ under a fixed compression force 0.003N are shown in **d** and **e**. The dependences of the axial displacement and the twisting angle on the applied force F are shown in **f** and **g**. γ range from 0 to 90º.

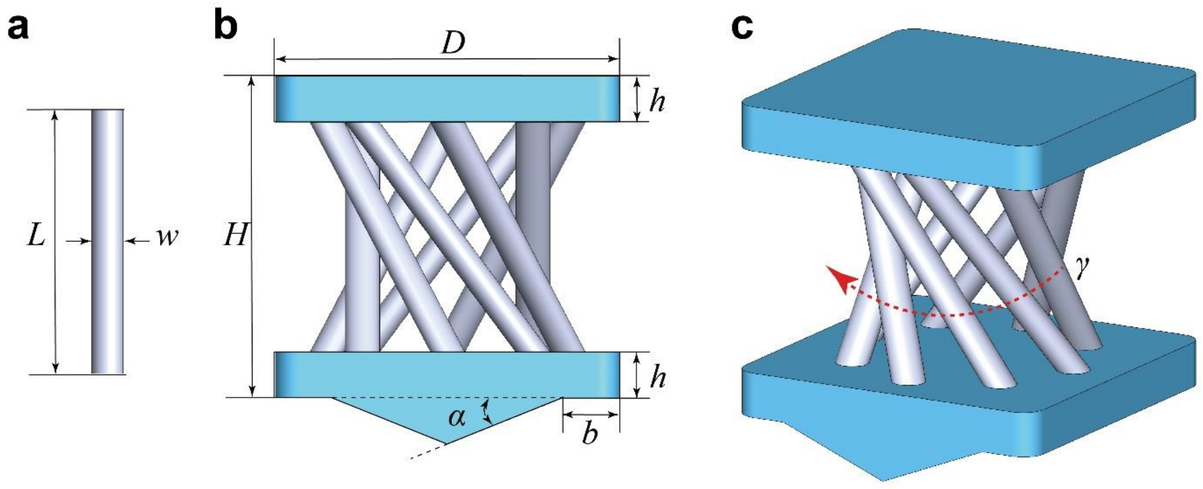

Supplementary Fig. 2. The design parameters of the chiral lattice foot. a The geometric parameters of a single rod. **b** The geometric parameters of the chiral lattice foot. **c** A left-handed chiral lattice foot with a rotation angle γ.

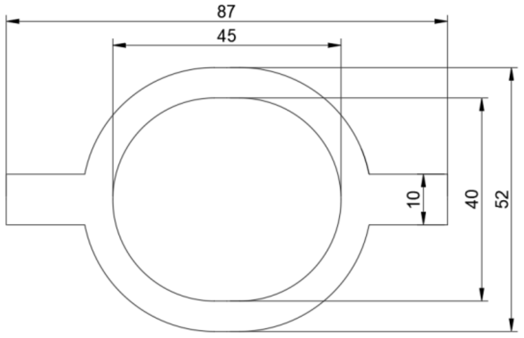

Supplementary Fig. 3. The CAD design of the acrylic frame. The unit is mm. The thickness of the acrylic sheet is generally 0.3mm.

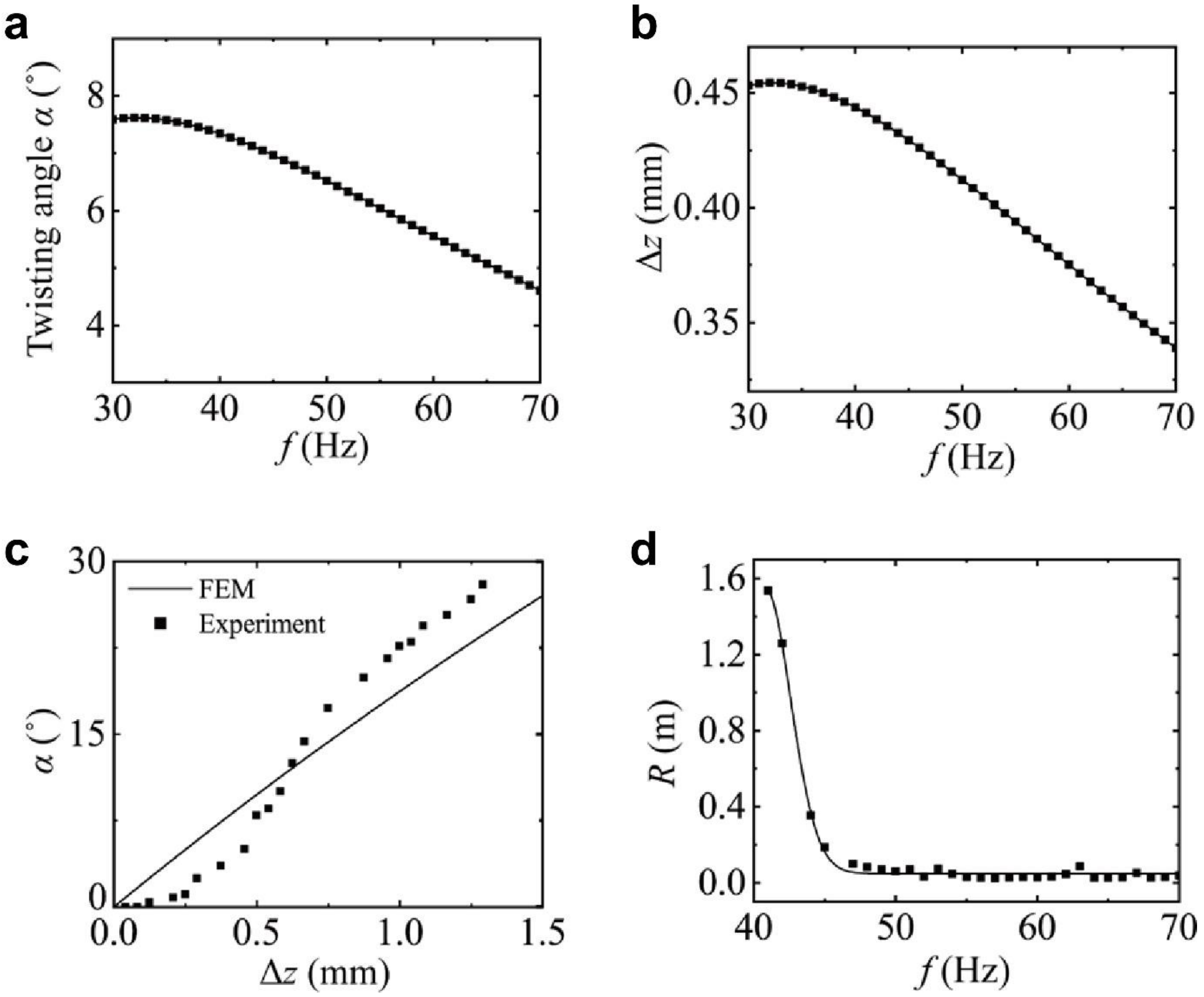

Supplementary Fig. 4. Quantitative results of the chiral lattice foot. a The dependence of the largest twisting angle α under different frequencies f. **b** The theoretically calculated maximum Δz at the steady state under various f. **c** FEM simulated and experimental α vs Δz. **d** The dependence of the experimentally measured radius of the circular path on f.

To measure the effective elastic constant of the lattice foot, we designed a bilayer lattice foot with symmetric shape, as shown in Supplementary Fig. 5a. A compression displacement is applied (Supplementary Fig. 5b, c). The strain rate is 1 mm/min. The experimentally and FEM compressive stress and strain relationship of a single lattice foot is shown in Supplementary Fig. 5d. The experimental linear fitted k is 35 N/m, 41 N/m and 72 N/m when the strain is 0.05 mm, 0.1 mm and 0.25 mm, respectively. When calculating the critical frequency of the bouncing behavior, the axial deformation of the lattice foot is small. We used an effective elastic constant $k = 38$ N/m, which is reasonable. Supplementary Fig. 5e shows the uniaxial tensile stress-strain curve of dog-bone specimens with Vero material. The Young's modulus of material is 1.5GPa which is obtained by linearly fitting the curves with a 3% strain. We choose 3% because the local strain of the material is generally less than 3% under compression.

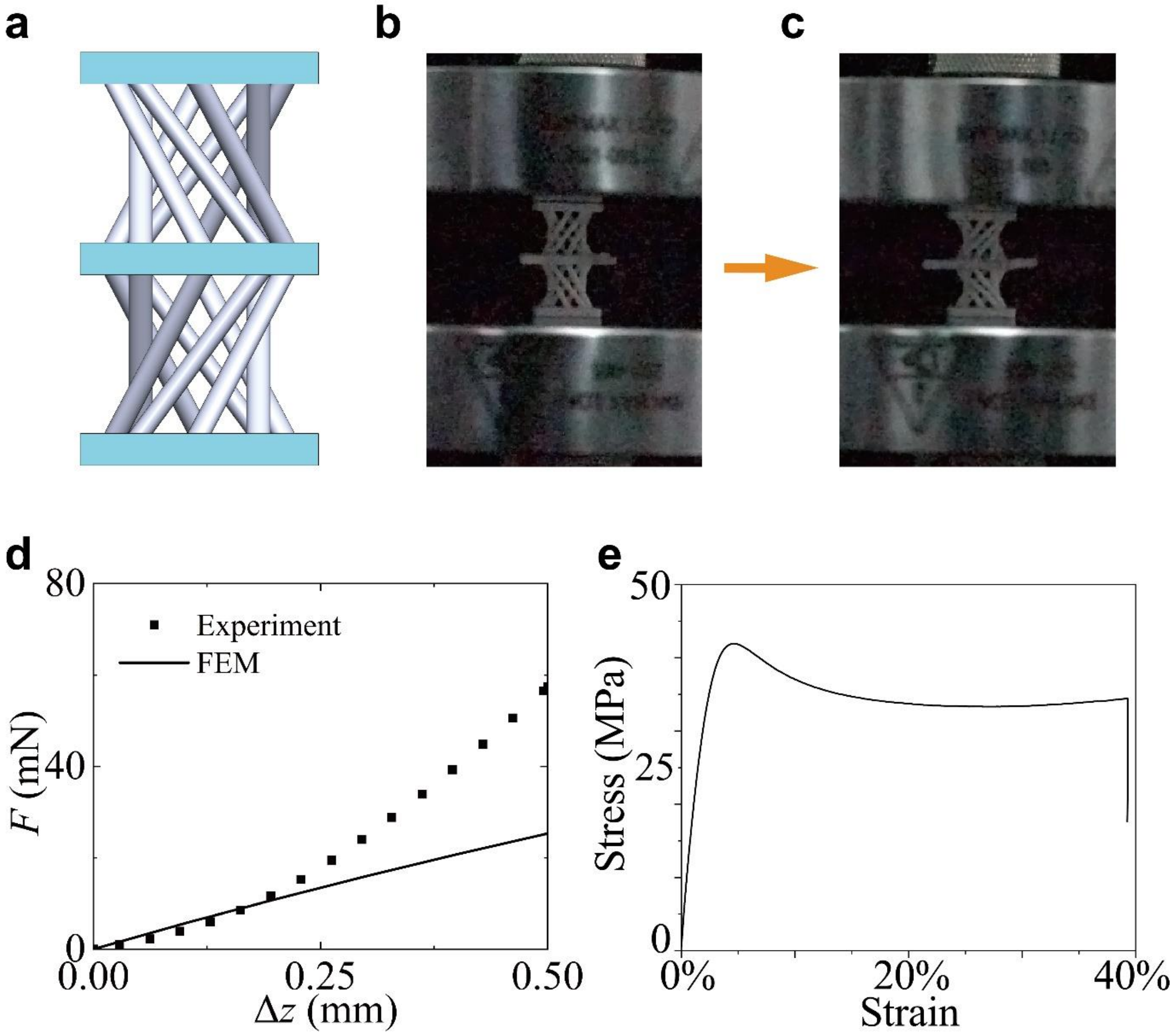

Supplementary Fig. 5. The dependence of the compressive force *F* and axial displacement *z*. a Structural design of a double layer chiral lattice foot. Experimental snapshots of the **b** undeformed and **c** deformed structures. **d** Comparisons of the finite element simulated and experimental compressive force F vs axial displacement Δz of the lattice foot. **e** Uniaxial tensile stress-strain curve of Vero material.

Supplementary Note 2. Locomotion Experiments

Supplementary Note 2.1. Displacement measurements

The experimental setup of the displacement measurement is shown in Supplementary Fig. 6a. The displacement of the soft robot is measured using a laser displacement sensor. The sampling frequency of the laser displacement is 1000Hz. The measured data under a 5kV,15Hz voltage is shown in Supplementary Fig. 6b.

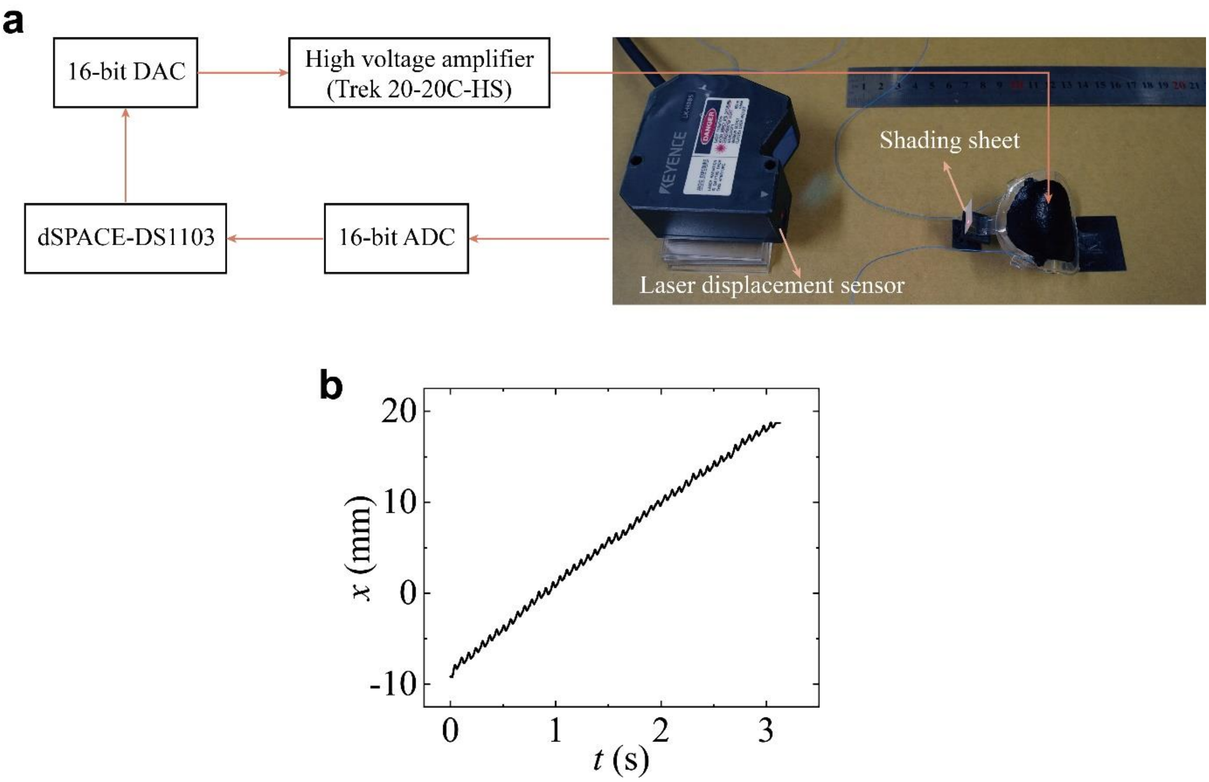

Supplementary Fig. 6. Displacement measurements. a The displacement of the soft robot is measured using a laser displacement sensor. The sampling frequency of the laser displacement is 1000Hz. **b** The measured data under a 5kV,15Hz voltage.

Supplementary Note 2.2. Blocking forces measurements

One end of the actuator is fixed on a plate, and the other is connected to a force sensor (Transform, K3D40) (Supplementary Fig. 7a). Initially, the soft robot is at the rest state, and the blocking force equals zero. We then apply a sinusoidal voltage and record the force sensor data using a high-voltage amplifier (TREK 10/10C-HS) and a dSPACE-DS1103 board. A 5kV sinusoidal voltage is applied with a frequency of 20 Hz, as shown in Supplementary Fig. 7b. The corresponding output force is shown in Supplementary Fig. 7c. Fourier transform of the output force is shown in Supplementary Fig. 7d. It can be seen that the primary peak frequency is 20 Hz, which is the same as the applied voltage. The output forces are also measured under an applied voltage of 6 kV and 7 kV at different frequencies from 1 to 45 Hz. The average maximum forces are calculated and shown in Supplementary Fig. 7e. The magnitude of the output force increases with the applied voltage and is not sensitive to the frequency. The phase differences between the output forces and applied voltages are measured (Supplementary Fig. 7f). The phase difference increases with the applied frequencies due to the viscoelastic properties of the VHB dielectric material.

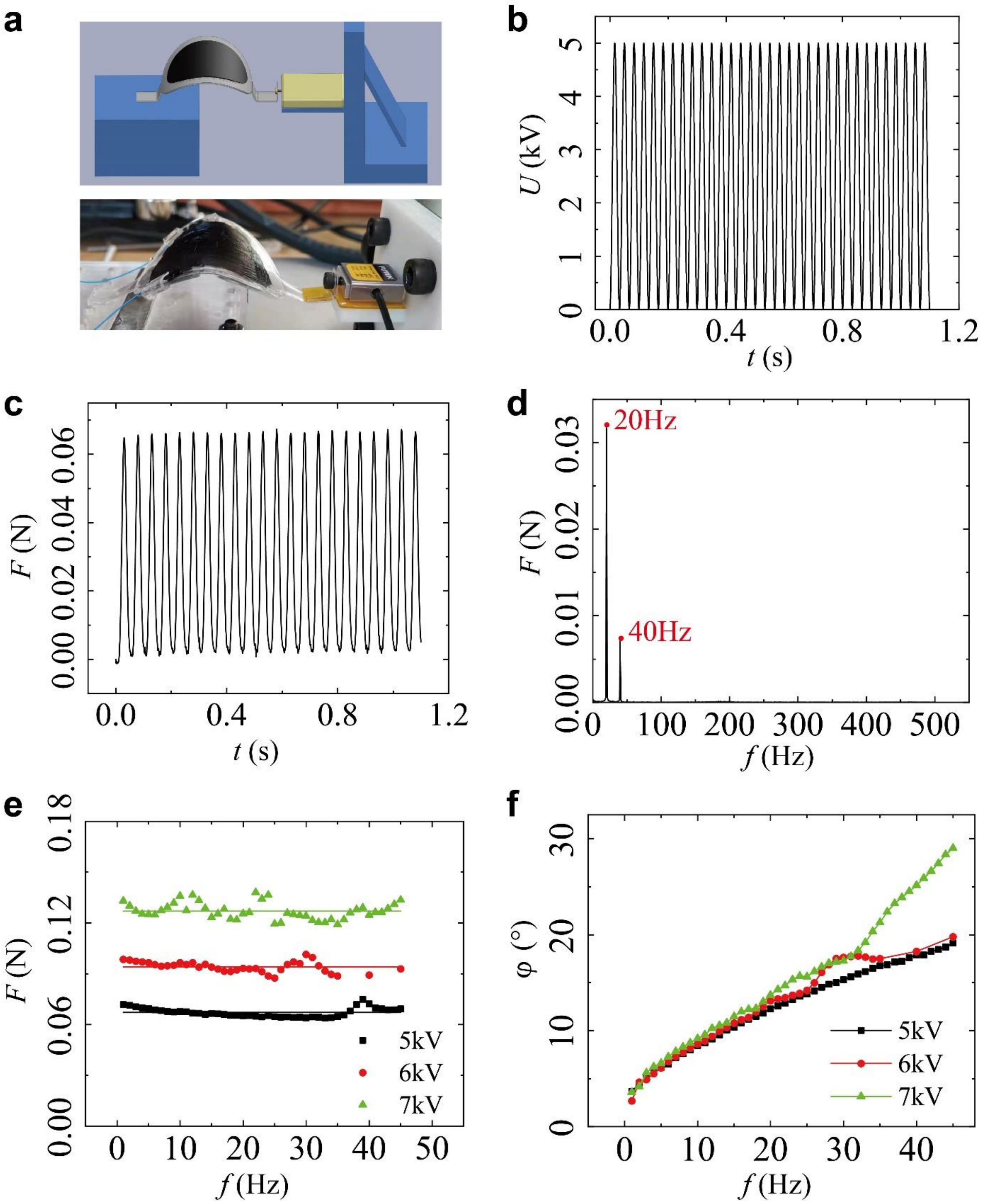

Supplementary Fig. 7. Blocking forces measurements. **a** Experimental setup. **b** A 5kV sinusoidal voltage is applied with a frequency of 20 Hz. **c** The corresponding output force. **d** Fourier transform of the output force. **e** The average maximum forces. **f** The phase differences between the output forces and applied voltages.

Supplementary Note 2.3 Cyclic tests

The breakdown voltage is around 8 kV. When the voltage exceeds this value, the DEA may break down by the high voltage and burn. The breakdown voltage depends on the deformed thickness of the DEA membrane. The thickness of the DEA is 1 mm, and the deformed area is 3.5×3.5 times the original area.

We have conducted cyclic tests of the saddled-shaped DE soft actuator using self-built equipment (Supplementary Fig. 8a). The left side of the soft actuator is attached to a platform driven by a motor. A cyclic displacement of +-3 mm is applied on the platform. The applied frequency is 30 Hz. The right side of the soft actuator is attached to a force sensor. Positive or negative force shows that the soft actuator is in expanded or contracted states. The average maximum and minimum forces during one minute are extracted for 6 hours with a 30 mins interval (Supplementary Fig. 8b). Supplementary Fig. 8c shows the measured force curves during one minute at the start, 2 hours later and 4 hours later. From the experimental results, we can observe that the soft robot remains relatively stable for 6 hours. The Fabrication procedures of the dielectric elastomer actuator is shown in Supplementary Fig. 9.

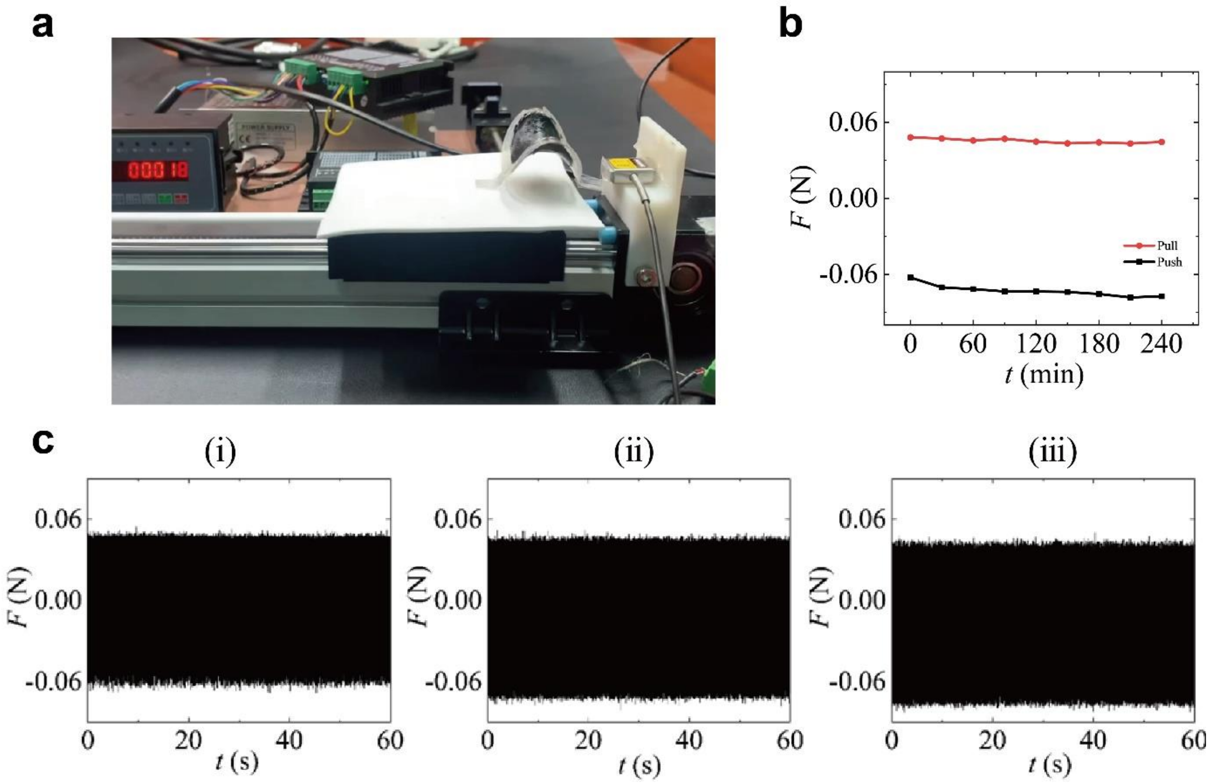

Supplementary Fig. 8. Cyclic tests of the DE actuator. **a** Experimental setup. **b** The average maximum and minimum forces during one minute for 6 hours with a 30 mins interval. **c** The measured forces in one minute at (i) start, (ii) 2 hours later and (iii) 4 hours later.

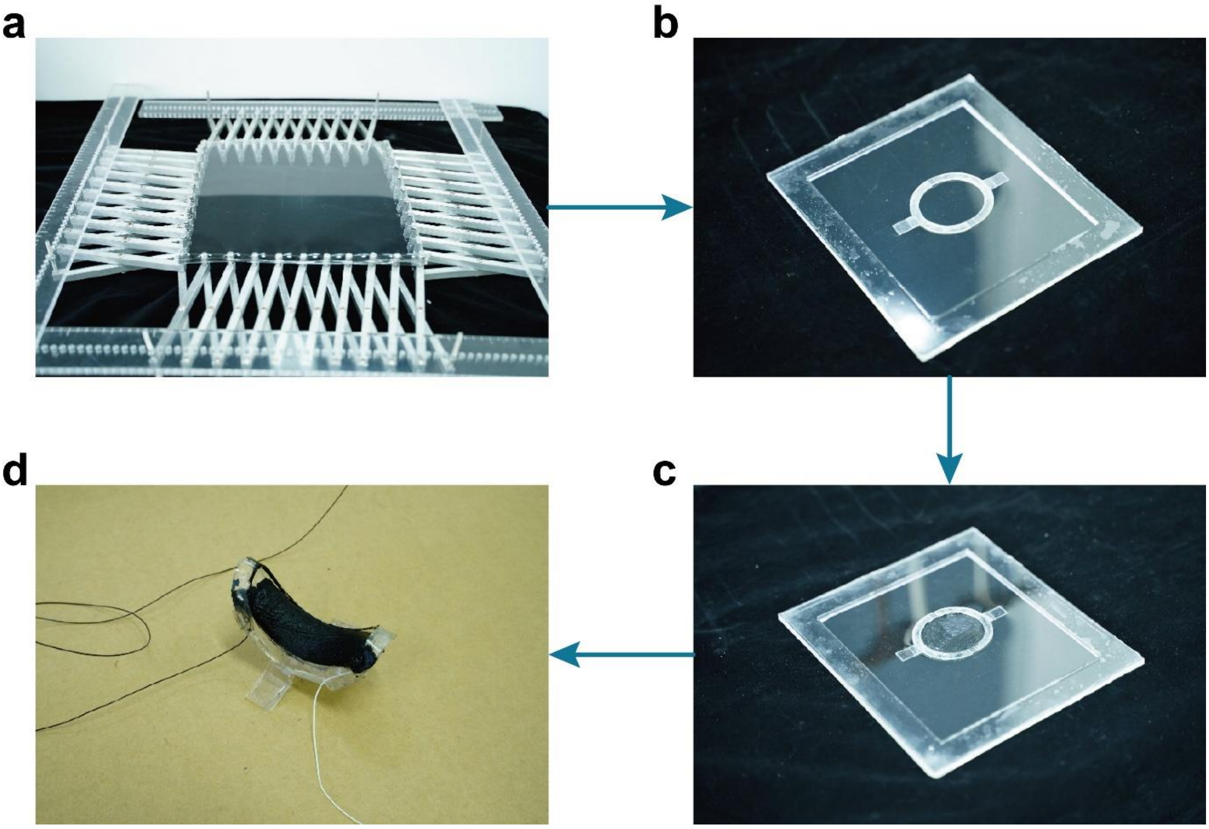

Supplementary Fig. 9. Fabrication procedures of the dielectric elastomer actuator. The dielectric elastomer actuator consists of a pre-stretched dielectric-elastomer membrane and a flexible acrylic frame. **a** The dielectric-elastomer membrane is biaxially pre-stretched 3.5×3.5 times. **b** The membrane is constraint by acrylic frame. **c** The dielectric-elastomer membrane is sandwiched by two compliant carbon grease electrodes. **d** After relaxing the prestretched membrane, the dielectric-elastomer actuator buckled into a saddle-shaped structure.

Supplementary Note 2.4. Motion change mechanism

Supplementary Fig. 10 shows the high-speed camera snapshot of the jumping of the lattice foot under a 35 Hz 6 kV voltage. The forward and backwards direction change is due to he jumping of the lattice foot. The bouncing diagram of the lattice foot at $\beta = 0.03$ is shown in Supplementary Fig. 11.

The turning mechanism is illustrated. The turning is controlled by the deviation angle, which is between the triangular prism and the vertical direction. One way to control the turning direction is by manually twisting the chiral lattice foot (Supplementary Fig. 12a and b). When the chiral lattice foot is twisted clockwise, the actuation force generated by the prism is perpendicular to the triangular prism and along the bottom right direction, which drives the soft robot to turn left (Supplementary Fig. 12a). Similarly, the soft robot turns right when the chiral lattice foot is twisted counter-clockwise (Supplementary Fig. 12b). The mechanism of turning right at high frequency is shown in Supplementary Fig. 12c. When the chiral lattice foot is compressed, the bottom plate rotates counter-clockwise, which drives the soft robot to turn

right.

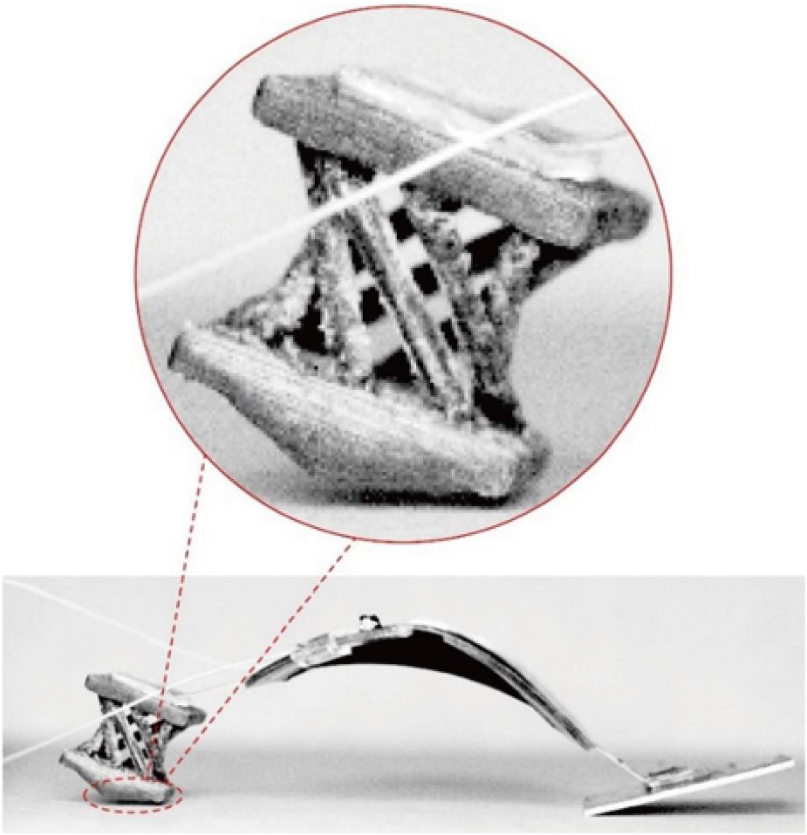

Supplementary Fig. 10. High-speed camera snapshot of the jumping of the lattice foot under a 35 Hz 6 kV voltage.

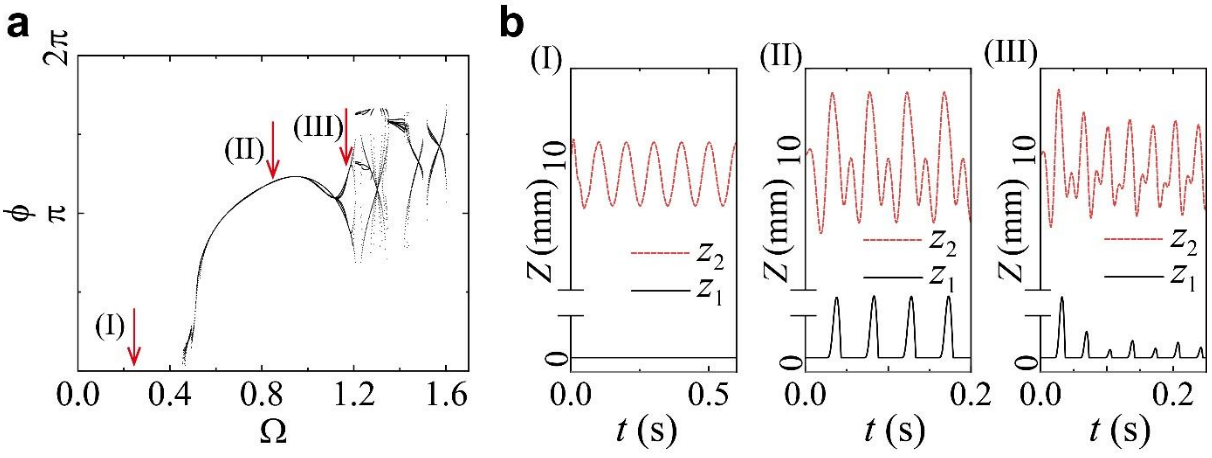

Supplementary Fig. 11. Bouncing diagram. a The bouncing diagram of the lattice foot at $\beta = 0.03$. The dimensionless flight time ϕ is shown as a function of Ω. **b** Three spatio-temporal diagrams showing the motions of the top (red) and bottom plates (black). The bottom plate stays on the ground at i, while it bounces once per two oscillations of the top plate at II and III.

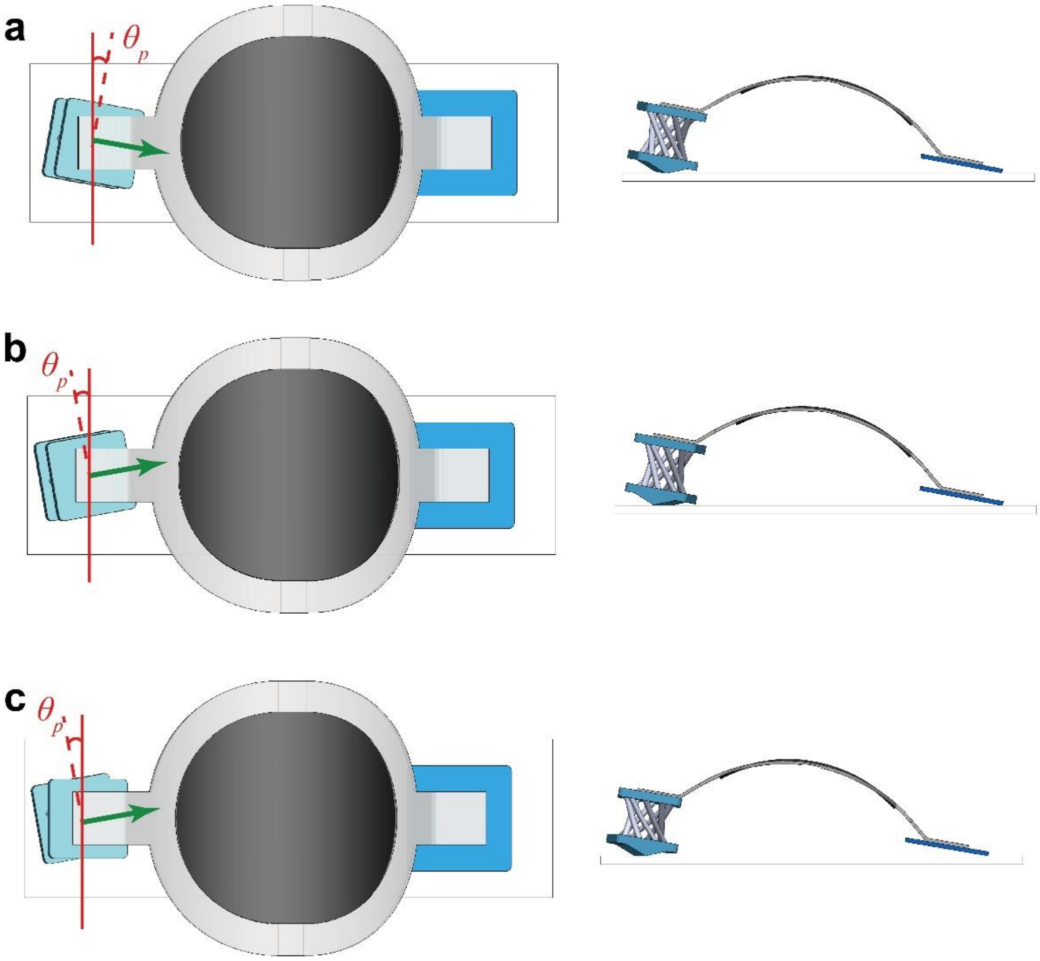

Supplementary Fig. 12. The direction of the prism of the chiral lattice foot. The top and front views are shown. a The soft robot turns left when the chiral lattice foot is twisted clockwise. **b** The soft robot turns right when the chiral lattice foot is twisted counter-clockwise. **c** When a left-handed chiral lattice foot is compressed, the bottom plate rotates counter-clockwise, and the soft robot turns right.

Supplementary Note 2.5. Effect of substrates

Results show that the substrate plays a vital role in the moving speed (Supplementary Movie 14). Five substrates are tested, as shown in Supplementary Fig. 13. The applied voltage is 6 kV. The moving speed is 7.7, 6.2, 3.4, 3.0, 0.4 mm/s when the substrate is rubber, paper, glass, acrylic and sandpaper, respectively. The friction ratios of the five substrates are around 0.6, 0.5, 0.4, 0.35 and 0.8. When the friction ratio is too small (acrylic and glass), both feet are slippery, and the moving speeds of the soft robots are small. When the friction ratio is too large (sandpaper), the actuation force cannot overcome the friction force, and the soft robot doesn't move. Substrates with suitable friction ratios are required for the soft robot.

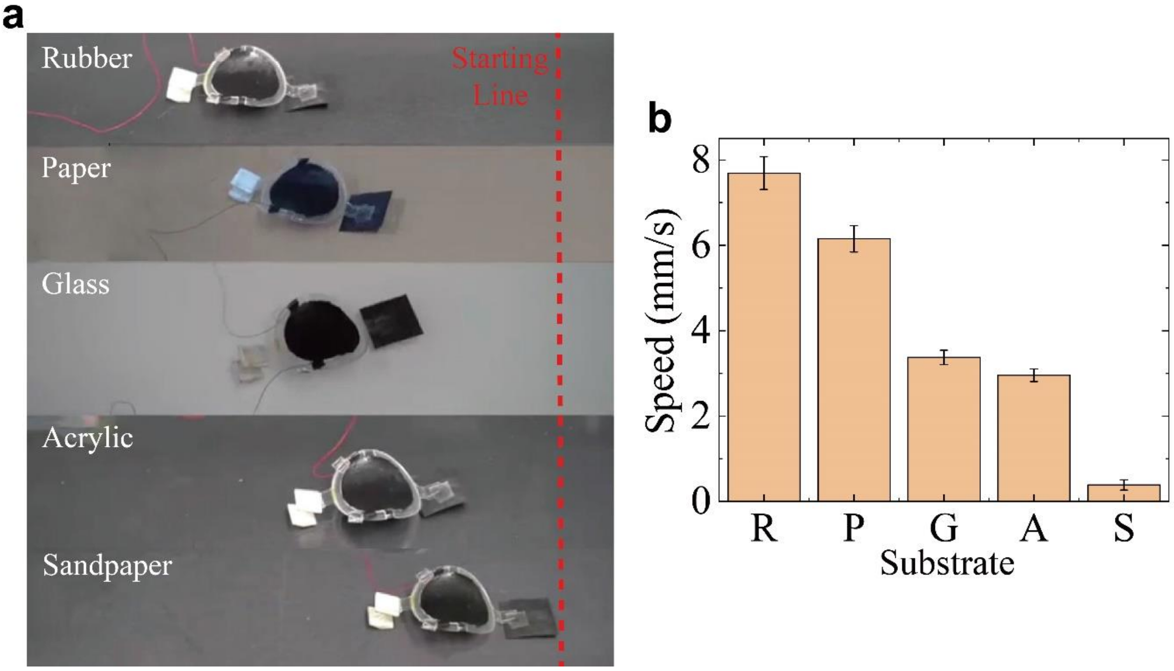

Supplementary Fig. 13. Effects of the substrates. a Five different substrates were used: rubber, paper, glass, acrylic and sandpaper. **b** The moving speed of the soft robots on various substrates.

Supplementary Note 2.6. Scalability

The soft robot is scalable (Supplementary Fig. 14, Movie 15 and 16). When the soft robot is scaled, the design parameters need to change correspondingly. The critical parameter is the thickness of the frame. When the frame is thick, the saddle shape is too flat to generate enough actuation. When the frame is thin, a saddle shape with large curvatures is formed, which leads to self-contact and causes the soft robot to burn. We have fabricated soft robots with 0.8 and 1.5 original sizes. The original acrylic frame is used for the 0.8-size soft robot, and we can observe that the soft robot can only move forward. Backwards or turning motion cannot be formed as the frame is thick. For the 1.5-size soft robot, we tried two different thicknesses, 0.4 mm and 0.5 mm. When the thin 0.4 mm framework is used, the DEA self-contact and burns under actuation. The soft robot can also move forward when the thick 0.5 mm framework is used. We measured the moving speed of the scaled soft robots. The forward speed of the 0.8-size soft robot is 4.6 mm/s. The forward speed of the 1.5-size soft robot is 4.6 mm/s. Their moving performances are not good, as the thickness of the acrylic frame is not optimal.

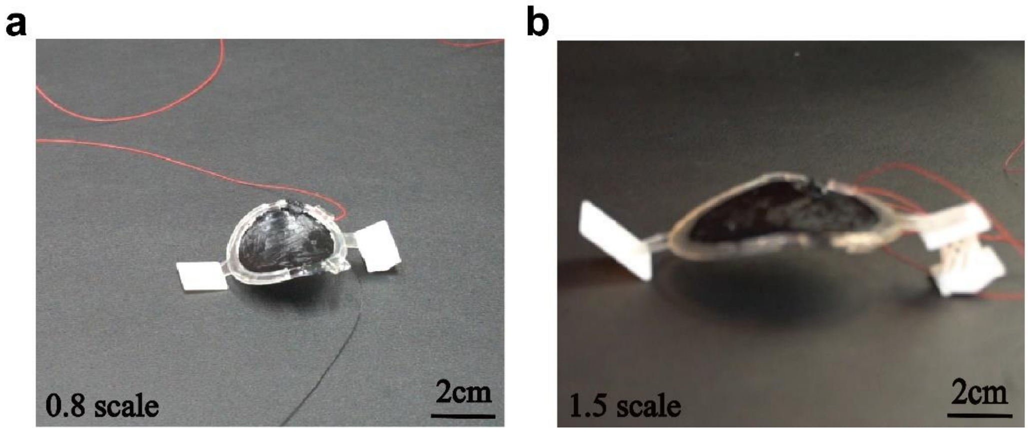

Supplementary Fig. 14. Scalability of the soft robots. Soft robots with **a** 0.8 and **b** 1.5 times the original size.

Supplementary Note 2.7. Carrying load

We have conducted experiments on the soft robot carrying a load. The soft robot can move forward, backwards or turn right when carrying a 2.2 g screw, which is around 46% of the soft robot's weight (4.8 g) (Supplementary Fig. 15a and Movie 18). When the load increases to 4.1g, the soft robot cannot move.

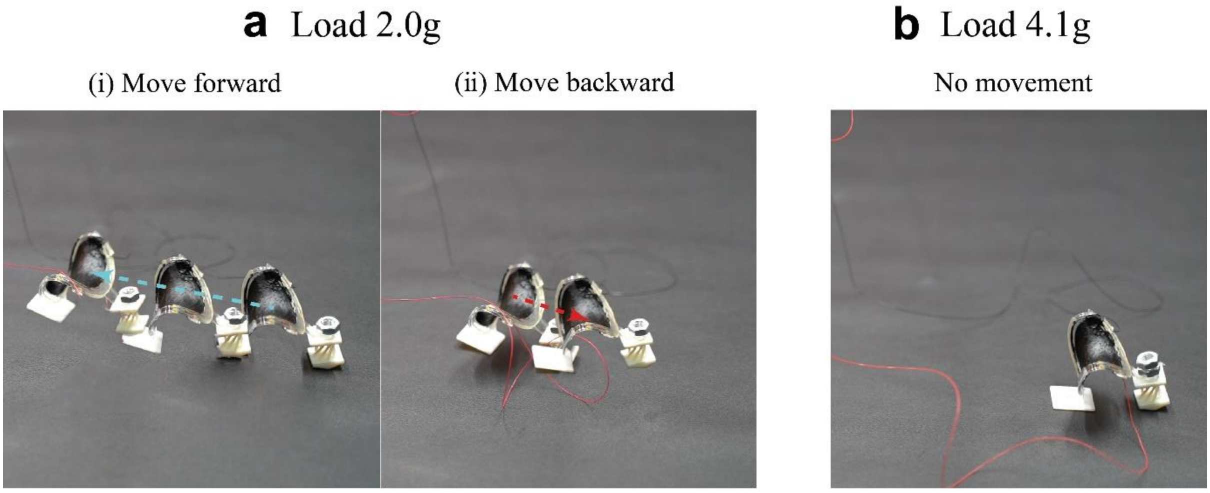

Supplementary Fig. 15. The performances of the soft robots carrying loads. a A load with 2.2g and **b** a load with 4.1g. The soft robot can move forward and backwards when the load is 2.2g. The soft robot cannot move when the load is 4.1g.

Supplementary Note 3. Shape reconfiguration

Supplementary Note 3.1. Switch time

The switch from left to right-hand circular motion is around 2 mins. The temperature is around 90 ºC. The switch time includes both the heating and cooling time. We have experimentally measured the transition time upon heating under various temperatures (Supplementary Fig. 16).

The material of the chiral lattice foot is a shape memory polymer with a glass transition temperature of ~58 ºC. The transition time change from ~22 s to ~1 s when T = 50ºC to 95ºC. The cooling time is relatively longer. Embedding cooling devices effectively reduces the cooling time, which we will explore as future work.

Supplementary Note 3.2. Multiple circles

After going one circle S, it is impossible to reheat again to return to the original circular as the Vero material is a one-way shape memory polymer. The lattice foot can only transform from the programmed state to the original state upon heating, but not the reverse. If a two-way shape memory polymer is used, it is possible to realize multiple S shapes. We can also use structural design to achieve two circles (Supplementary Movie 12). We design a bilayer lattice foot, as shown in Supplementary Fig. 17a. Both layers are programmed first (Supplementary Fig. 17b). Under an electric actuation with f = 45 Hz, the soft robot turns right (A to B in Supplementary Fig. 17e). After it reaches point B, a thermal stimulus is applied to the bottom layer of the foot, and it recovers to its original shape (Supplementary Fig. 17c). The triangular prism forms an angle with the centerline of the soft robot due to the twist of the bottom layer, which drives the soft robot to turn left from B to C. We then heat the top layer, and the prism changes to the left direction. The soft robot turns right (C to D). We need to mention that the above design method is unstable due to heat conduction. A stable way to realize a complex trajectory is by applying different frequencies. We have controlled the soft robot to explore a maze by varying the frequency to show its controllability, as shown in Supplementary Movie 13.

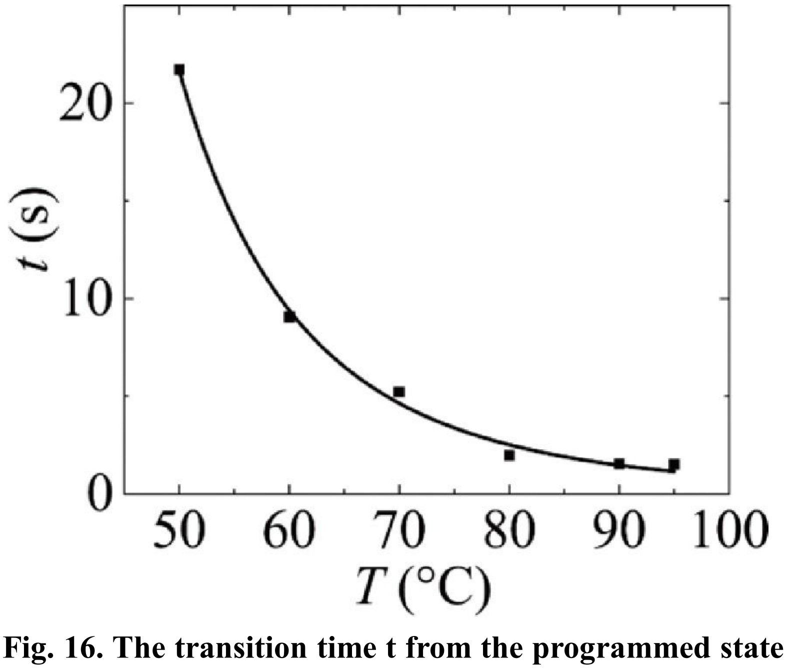

Supplementary Fig. 16. The transition time t from the programmed state to the original shape at various temperatures *T*.

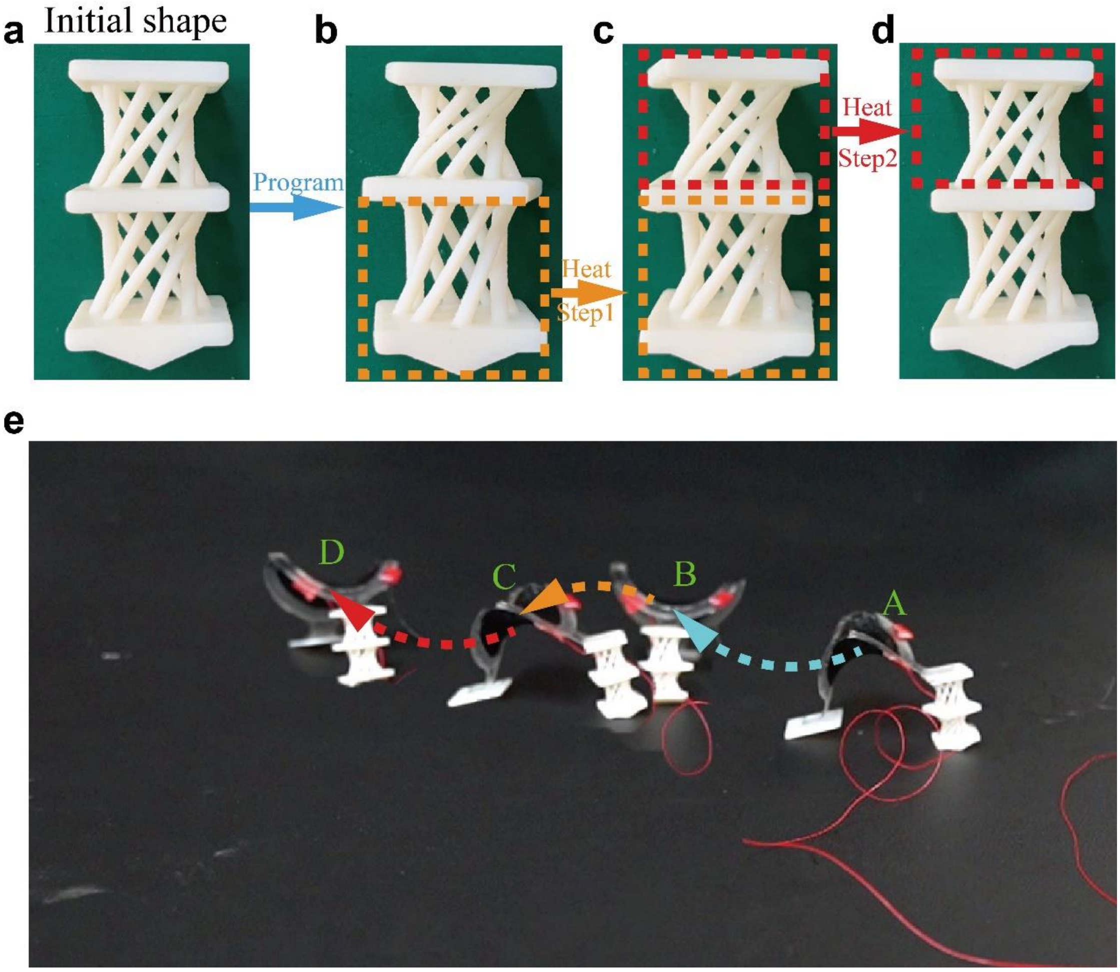

Supplementary Fig. 17. A soft robot with bilayer chiral lattice foot. a The initial shape, **b** programmed state, **c** recovered state I and **d** recovered state II of a bilayer lattice foot. **e** The moving trajectory of the soft robot.

Supplementary Note 3.3. Integrated heating element

We used a ceramic heating plate as the heating element (Supplementary Fig. 18 and Movie 17). The structure of the chiral foot is shown in Supplementary Fig. 18b. An external DC power with 5V and 1A current is used to heat the feet. Supplementary Fig. 18a shows the moving trajectory of the soft robot with an embedded heater. The soft robot turns right first under a frequency of 45 Hz. We then use external heat to heat the feet. Upon heating, the foot rotates and recovers to its original shape, which forms an angle with the central line of the soft robot. The inclined angle drives the soft robot to turn left.

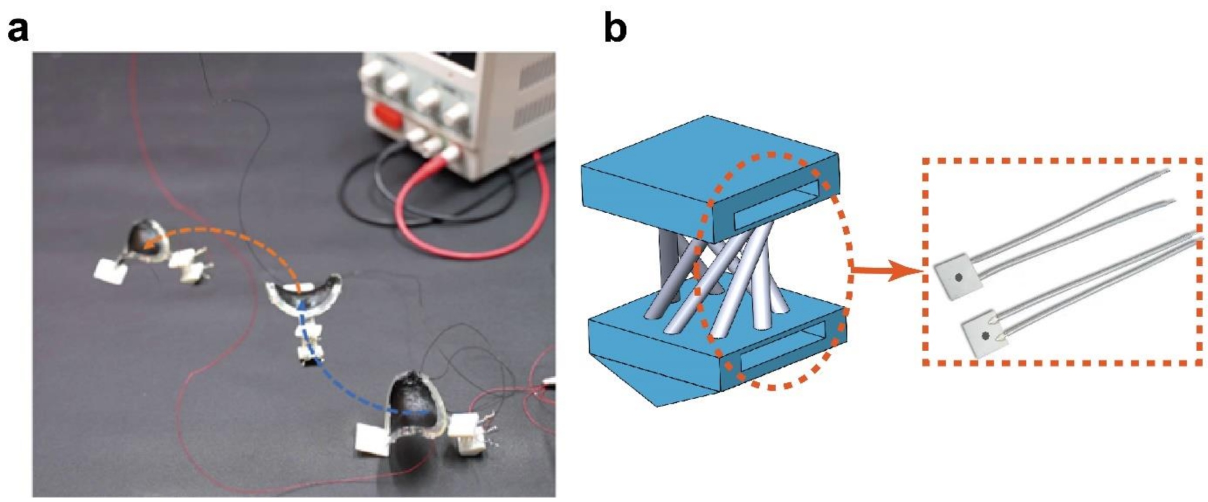

Supplementary Fig. 18. Locomotion of a soft robot with embedded heater. a The moving trajectory. **b** The structural design of the chiral foot.

Supplementary Note 4. Soft robots with other types of feet

In Supplementary Fig. 19a, the soft robot uses two symmetric feet. With the symmetric feet, the soft robot exhibit contraction or extension with respect to its center under applied voltage. Hence, the soft actuator remains in its original position. Using asymmetric feet, a net force is generated that converts the stored elastic energy into locomotion. Three types of asymmetric feet are used: rectangular, round and sawtooth asymmetric feet (Supplementary Fig. 19b-d). The geometries of the asymmetric feet are shown beside. Two materials with different friction ratios are used to fabricate the lattice feet: Agilus and VeroClear. The friction ratios of the two materials are tested as 0.28 for VeroClear and 0.5 for Agilus on a cloth substrate[1]. The feet are fabricated using a commercial Polyjet 3D printer J750. It can be seen that by using simple rectangular or round asymmetric feet, the soft robot moves in the right direction. However, the motion is stable. By using the sawtooth feet, the movement is much more stable.

Besides the asymmetry generated using materials with different friction ratios, asymmetry shapes are also used to induce locomotion. As shown in Supplementary Fig. 19e, two wedge-shaped feet are used. The wedged shape is asymmetric around its center. Therefore, asymmetric frictions are generated and lead to locomotion. In Supplementary Fig. 19f, the left wedge-shaped foot is rotated by 30 degrees. Consequently, the soft robot jumps as it moves, and the moving speed increases significantly from 13 mm/s to 20 mm/s.

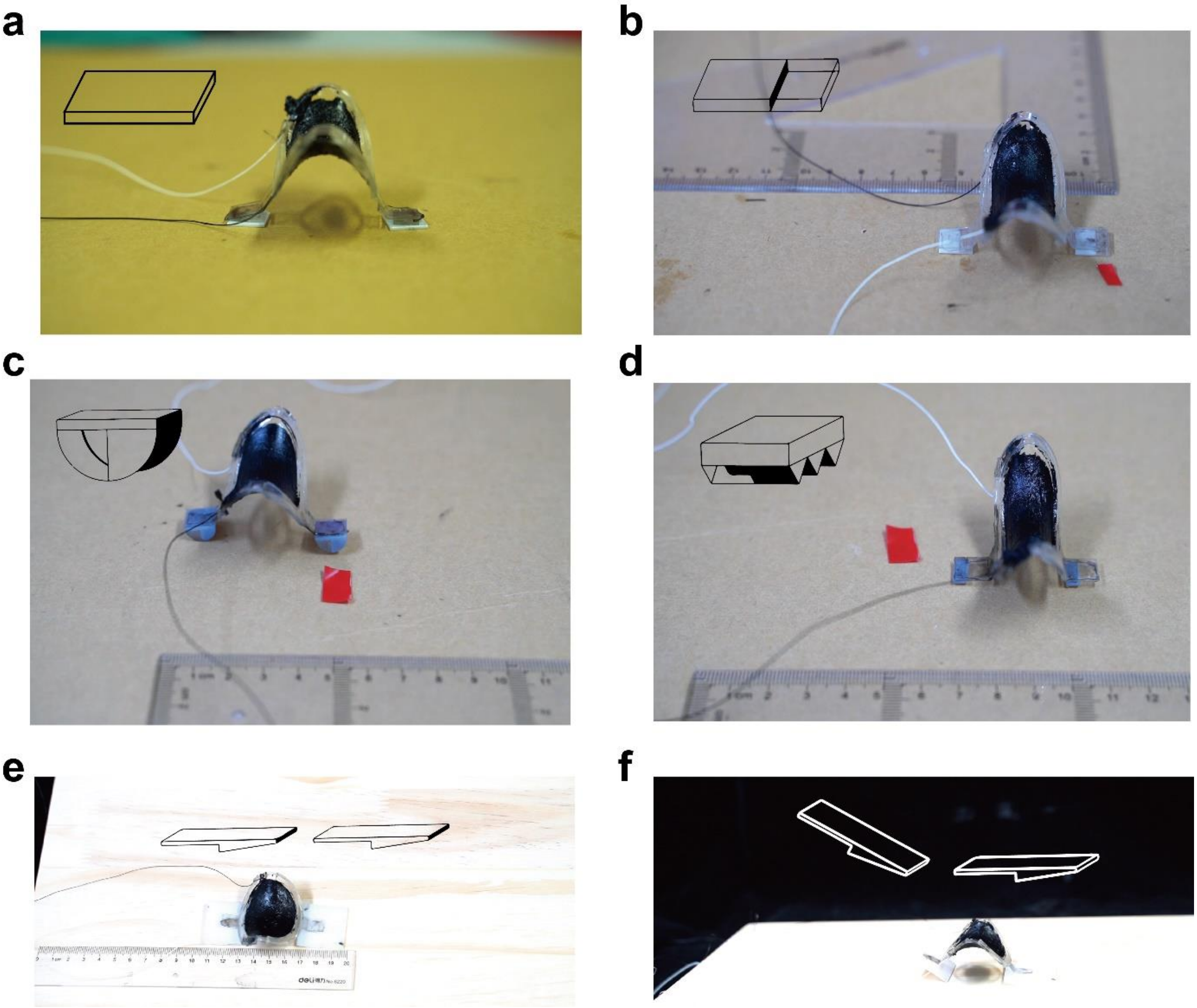

Supplementary Fig. 19. Experimental study of the locomotion of soft robots using six different types of feet. The key to producing locomotion is to break the self-symmetry. Self-symmetry generally leads to a contraction or extension to its center, while breaking the symmetry can generate a net gain that converts the stored elastic energy into translational motion. **a** a pair of symmetric rectangular feet. **b** Rectangular feet with asymmetric material distribution. **c** circular feet with asymmetric material distribution. **d** Sawtooth feet with asymmetric material distribution. **e** Wedge-shaped feet. **f** Wedge-shaped feet with different titled angles.

Table S1. Soft crawling robots with direction change ability.

Soft Robots	Actuation	Maximum speed	Direction changeable	Response to external stimuli
Mihai Duduta, 2017[2]	DE	1.03BL/s	No	No
Yichao Tang, 2020[3]	Pneumatic	2.68 BL/s 0.78BL/s(underwater)	No	No
Yichuan Wu, 2019[4]	PVDF	20 BL/s	No	No
Guoying Gu, Jiang Zou, 2018[5]	DE	1.04 BL/s	Yes	No
Jiawei Cao, 2018[6]	DE	0.02 BL/s	Yes	No
Guorui Li, 2021[7]	DE	0.34BL/s	No	No
Tiefeng Li, 2017[8]	DE	0.69BL/s	No	No
Canh Toan Nguyen, 2017[9]	DE	0.2BL/s	No	No
Haojian Lu, 2018[10]	Magnetic	0.029BL/s	No	No
Xiaobin Ji, 2019[11]	DE	0.75BL/s	Yes	No
Dylan Drotman, 2021[12]	Pneumatic	0.05BL/s	Yes	Yes
Dohgyu Hwang, 2022[13]	Water jet propulsion	1.3BL/s	No	Yes
Dae-Young Lee, 2017[14]	Motor	1.1BL/s	No	Yes
Dylan S, 2020[15]	Pneumatic	0.23BL/s	Yes	Yes
Chao Tang, 2022[16]	DE	1.19BL/s	Yes	No
Huachen Cui, 2022[17]	Electric field	6.4BL/s	Yes	Yes

References:

1 Sheng, X. *et al.* Multi-material 3D printing of caterpillar-inspired soft crawling robots with the pneumatically bellow-type body and anisotropic friction feet. *Sens. Actuator A Phys.* **316**, 112398 (2020).

2 Duduta, M., Clarke, D. R. & Wood, R. J. in *2017 IEEE International Conference on Robotics and Automation (ICRA).* 4346-4351 (IEEE).

3 Tang, Y. *et al.* Leveraging elastic instabilities for amplified performance: Spine-inspired high-speed and high-force soft robots. *Sci. Adv.* **6**, eaaz6912 (2020).

4 Wu, Y. *et al.* Insect-scale fast moving and ultrarobust soft robot. *Sci. Robot.* **4**, eaax1594 (2019).

5 Gu, G., Zou, J., Zhao, R., Zhao, X. & Zhu, X. Soft wall-climbing robots. *Sci. Robot.* **3** (2018).

6 Cao, J. *et al.* Untethered soft robot capable of stable locomotion using soft electrostatic actuators. *Extreme Mech. Lett.* **21**, 9-16 (2018).

7 Li, G. *et al.* Self-powered soft robot in the Mariana Trench. *Nature* **591**, 66-71 (2021).

8 Li, T. *et al.* Fast-moving soft electronic fish. *Sci. Adv.* **3**, e1602045 (2017).

9 Nguyen, C. T., Phung, H., Nguyen, T. D., Jung, H. & Choi, H. R. Multiple-degrees-of-freedom dielectric elastomer actuators for soft printable hexapod robot. *Sens. Actuator A Phys.* **267**, 505-516 (2017).

10 Lu, H. *et al.* A bioinspired multilegged soft millirobot that functions in both dry and wet conditions. *Nat. Commun.* **9**, 1-7 (2018).

11 Ji, X. *et al.* An autonomous untethered fast soft robotic insect driven by low-voltage dielectric elastomer actuators. *Sci. Robot.* **4**, eaaz6451 (2019).

12 Drotman, D., Jadhav, S., Sharp, D., Chan, C. & Tolley, M. T. Electronics-free pneumatic circuits for controlling soft-legged robots. *Sci. Robot.* **6**, eaay2627 (2021).

13 Hwang, D., Barron III, E. J., Haque, A. T. & Bartlett, M. D. Shape morphing mechanical metamaterials through reversible plasticity. *Sci. Robot.* **7**, eabg2171 (2022).

14 Lee, D.-Y., Kim, S.-R., Kim, J.-S., Park, J.-J. & Cho, K.-J. Origami wheel transformer: A variable-diameter wheel drive robot using an origami structure. *Soft Robot.* **4**, 163-180 (2017).

15 Shah, D. S. *et al.* A soft robot that adapts to environments through shape change. *Nat. Mach. Intell.* **3**, 51-59 (2021).

16 Tang, C. *et al.* A pipeline inspection robot for navigating tubular environments in the sub-centimeter scale. *Sci. Robot.* **7**, eabm8597 (2022).

17 Cui, H. *et al.* Design and printing of proprioceptive three-dimensional architected robotic metamaterials. *Science* **376**, 1287-1293 (2022).
