# Supplementary material for: Dexterous electrical-driven soft robots with reconfigurable chiral-lattice foot design
Source: Nat Commun. 2023 Aug 21;14:5067. doi: 10.1038/s41467-023-40626-x (PMC10442442; doi:10.1038/s41467-023-40626-x)
Supplement: Supplementary file 3 — Description of Additional Supplementary Files [file 41467_2023_40626_MOESM3_ESM.pdf]

Description of Additional Supplementary Files File

Name: Supplementary Movie 1
Description: Theoretically predicted bouncing behaviors of the lattice foot under f = 10, 30 and 50 Hz.

Name: Supplementary Movie 2
Description: Forward and backward locomotion. The soft robot moves to the right at f = 30 Hz, and moves to the left at f = 15 Hz.

Name: Supplementary Movie 3
Description: Movement of the soft robots captured by a high-speed camera at frequencies of 15 Hz, 23 Hz and 30 Hz.

Name: Supplementary Movie 4
Description: Theoretically predicted bouncing behaviors of the lattice foot under f = 46 Hz, 7kV.

Name: Supplementary Movie 5
Description: The soft robot moves circularly at f = 46 Hz.

Name: Supplementary Movie 6
Description: The soft robot can reach four points A, B, C and D in the four quadrants by adjusting the frequency.

Name: Supplementary Movie 7
Description: The soft robot can move in the left or right circular directions under f = 30 Hz by adjusting the orientations of the lattice foot.

Name: Supplementary Movie 8
Description: The soft robot can form an S-shaped trajectory using the shape memory effect of the lattice foot.

Name: Supplementary Movie 9
Description: The soft robot can pass through a narrow tunnel.

Name: Supplementary Movie 10
Description: Locomotion behaviors of the soft robot using other feet.

Name: Supplementary Movie 11
Description: The soft robot performs a stationary turn under a 54 Hz voltage.

Name: Supplementary Movie 12
Description: The soft robot that moves in a multiple circles trajectory.

Name: Supplementary Movie 13
Description: The soft robot explore a maze by varying the frequency (4×speed).

Name: Supplementary Movie 14
Description: The speed performance of a soft robot varies on different types of substrates.

Name: Supplementary Movie 15
Description: Locomotion of a 1.5-size soft robot.

Name: Supplementary Movie 16
Description: Locomotion of a 0.8-size soft robot.

Name: Supplementary Movie 17
Description: Locomotion of a soft robot with embedded heater.

Name: Supplementary Movie 18
Description: The performances of the soft robots carrying loads.
